# Exploring the impact of urban spatial morphology on land surface temperature: A case study in Linyi City, China

**DOI:** 10.1371/journal.pone.0317659

**Published:** 2025-01-27

**Authors:** Yongyu Feng, Huimin Wang, Jing Wu, Yan Wang, Hui Shi, Jun Zhao

**Affiliations:** Science and Technology Innovation Team of Shandong Provincial Department of Natural Resources, Shandong Institute of Land Spatial Data and Remote Sensing Technology, Jinan, China; AUM: American University of the Middle East, KUWAIT

## Abstract

The increasing population density and impervious surface area have exacerbated the urban heat island effect, posing significant challenges to urban environments and sustainable development. Urban spatial morphology is crucial in mitigating the urban heat island effect. This study investigated the impact of urban spatial morphology on land surface temperature (LST) at the township scale. We proposed a six-dimensional factor system to describe urban spatial morphology, comprising Atmospheric Quality, Remote Sensing Indicators, Terrain, Land Use/Land Cover, Building Scale, and Socioeconomic Factors. Spatial autocorrelation and spatial regression methods were used to analyze the impact. To this end, the township-scale data of Linyi City from 2013 to 2022 were collected. The results showed that LST are significantly influenced by urban spatial morphology, with the strongest correlations found in the factors of land use types, landscape metrics, and remote sensing indices. The global Moran’s I value of LST exceeds 0.7, indicating a strong positive spatial correlation. The High-High LISA values are distributed in the central and western areas, and the Low-Low LISA values are found in the northern regions and some scattered counties. The Geographically Weighted Regression (GWR) model outperforms the Spatial Error Model (SEM) and Ordinary Least Squares (OLS) model, making it more suitable for exploring these relationships. The findings aim to provide valuable references for town planning, resource allocation, and sustainable development.

## 1. Introduction

Land Surface Temperature (LST) is a critical factor influencing energy exchange [1] and water cycle [2] processes between the land and the atmosphere, and it is a significant indicator of surface energy balance [3]. China, as one of the countries experiencing rapid urbanization, has seen a swift increase in urbanization rates [4]. The surge in urban population and extensive development has led to significant environmental challenges [5], particularly the urban heat island (UHI) effect [6], severely impacting the urban ecological environment and sustainable development [7]. Thus, understanding the influence of urban spatial morphology on LST in urban zones is of paramount importance for addressing the widely concerning UHI issue [8].

Currently, numerous scholars employ remote sensing and meteorological data to study LST and the UHI effect in specific research areas [9–14]. These studies focus on data sources, unit selection, analytical methods, driving factors, mitigation measures, and effective simulations. However, the relationship between the observed land surface temperature (LST) and surface cover structure is blurred due to the limited spatial resolution of remote sensing images. Therefore, it is very important to choose appropriate analysis units and factors to analyze the characteristics of LST.

Statistical and linear analysis models with data from remote sensing and meteorological sources were used for quantitative analysis of temporal and spatial factors [15]. Temporal factors primarily focus on LST variations within fixed periods (e.g., year, season, day or night) [16]. Spatial factors include urban three-dimensional or two-dimensional forms [17]. However, existing studies on LST relationships mainly conduct quantitative analyses of the natural environment, meteorological, and anthropogenic factors, often neglecting the systematic spatial morphology of cities [18,19]. Moreover, the mechanisms of LST spatial distribution impacts on UHI are mainly studied at the urban scale overlooking urban spatial heterogeneity [20], limiting the applicability of research findings at local scales [21].

The term "urban morphology" refers to the spatial arrangement and structure of the urban landscape, which is widely acknowledged as a primary factor influencing the UHI effect due to its close association with urban heat transfer and ventilation processes. This study, emphasizing regional complexity from a geographical perspective, constructs an urban spatial morphology factor system. Utilizing spatial autocorrelation analysis and spatial econometric models, it identifies and quantifies the impact of urban spatial morphology on LST. Linyi City, as a typical inland city with unique geographical environment and thermal characteristics, serves as the research subject. The study has three objectives: (a) to construct an urban spatial morphology factor system using six types of factors—Atmospheric Quality, Remote Sensing Indicators, Terrain, Land Use/Land Cover, Building Scale, and Socioeconomic Factors; (b) to explore the spatiotemporal patterns of LST using spatial autocorrelation methods; and (c) to investigate the relationship between LST and urban spatial morphology using the Geographically Weighted Regression method.

## 2. Materials and methods

### 2.1 Study area

**Fig 1:**
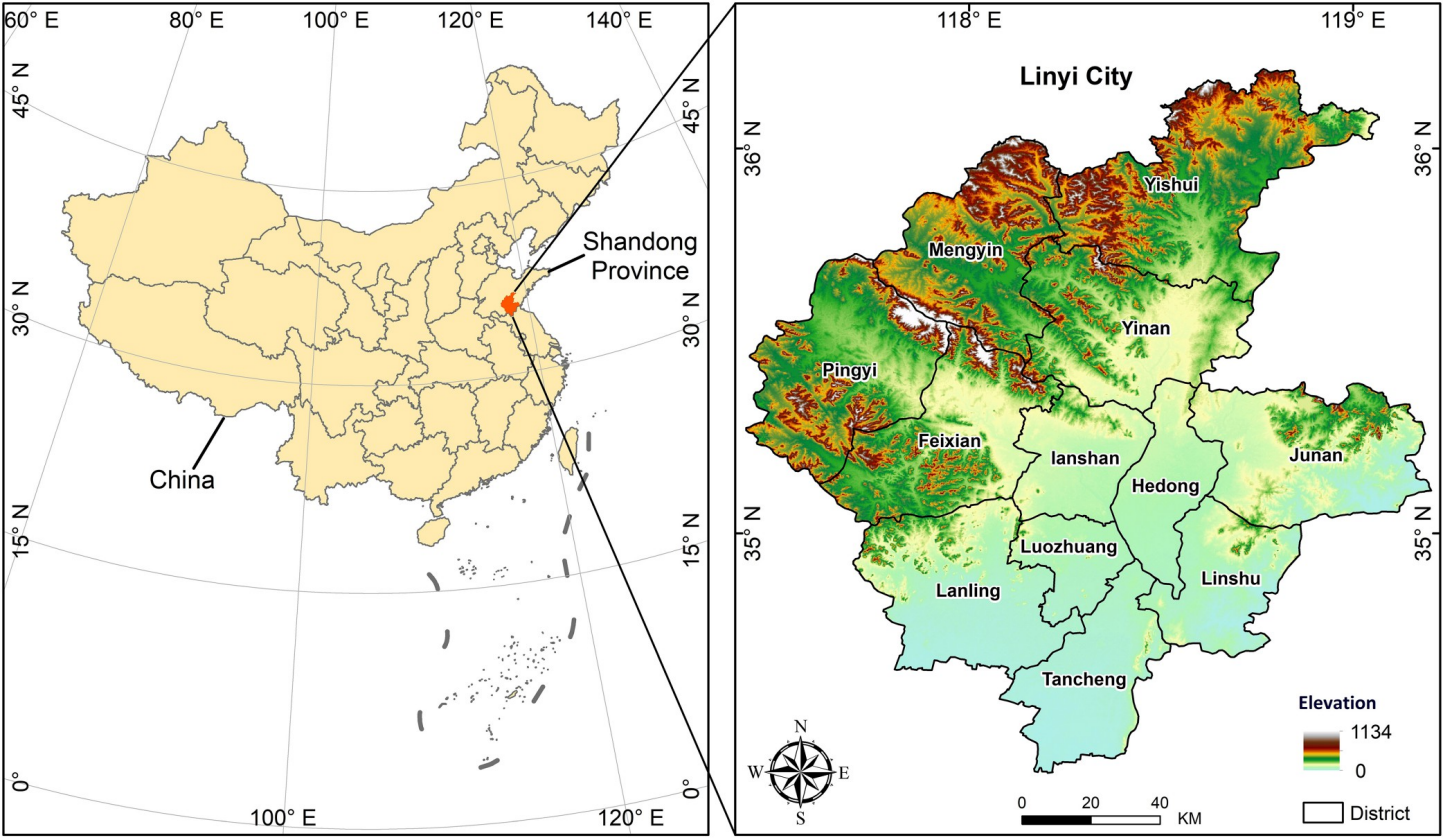
Location and study area (Linyi City, Shandong Province, China). USGS EROS (Earth Resources Observatory and Science (EROS) Center) (public domain): http://eros.usgs.gov/.

Linyi City, located in the southern part of Shandong Province, eastern China, is a significant logistics hub. The city comprises 12 districts and counties with a total of 156 townships: Lanshan District (12 townships), Luozhuang District (9 townships), Hedong District (11 townships), Yinan County (15 townships), Tancheng County (13 townships), Yishui County (18 townships), Lanling County (17 townships), Feixian County (12 townships), Pingyi County (14 townships), Junan County (16 townships), Mengyin County (9 townships), and Linshu County (10 townships). The city’s terrain is a mix of mountains, hills, and plains, with mountainous and hilly areas in the northwest and flat plains in the southeast (Fig 1). The climate is a warm temperate monsoon type, characterized by cold winters, hot summers, and abundant rainfall. As the third-largest city in Shandong Province, Linyi has substantial transportation, agricultural, and population resources.

The expanding urban size has increased the demand for land and natural resources, leading to severe pollutant emissions [22]. With the city’s comprehensive strength continuously improving, the urban spatial pattern and functional zoning are evolving, significantly impacting the urban heat island effect [23]. Townships, as vital administrative units in urban management in China, can respond to various urban development strategies [24]. Changes in the spatial functions of townships can significantly affect the management of the urban heat island effect [25]. This research investigates the impact of urban spatial morphology on LST at the township scale from 2013 to 2022 in Linyi City, providing valuable references for future urban spatial planning.

### 2.2 Data

#### 2.2.1 Data sources

The research dataset includes types such as remote sensing images, digital elevation models, land use, built-up area information, population, and urbanization levels (Table 1). MODIS imagery provides aerosol optical depth data (https://lpdaac.usgs.gov/products/mcd19a2v061/), reflecting urban air pollution conditions [26,27]. Landsat imagery provides surface reflectance data and digital elevation models (https://www.usgs.gov/centers/eros/science), covering a global scale [28,29]. Land use data are sourced from ESRI’s latest 10-meter resolution data released in 2022 (https://www.arcgis.com/home/item.html?id=cfcb7609de5f478eb7666240902d4d3d) [30,31]. Built-up area, population, and urbanization level data are obtained from the European Commission’s Global Human Settlement Layer (GHSL) (https://ghsl.jrc.ec.europa.eu/datasets.php) [32,33].

**Table 1:** Explanation of variables and data sources.

Type	Variable (abbreviation)	Data provider	Spatial resolution
Atmospheric quality	Aerosol Optical Depth (AOD)	NASA LP DAAC at the USGS EROS Center (public domain)	1000 m
Remote sensing indicators	Normalized Difference Vegetation Index (NDVI)	USGS EROS Center (public domain)	30 m
	Normalized Difference Water Index (NDWI)		
	Normalized Difference Built-up Index (NDBI)		
	Built-up Index (BU)		
Terrain	Elevation (HGT)	USGS EROS Center (public domain)	7.5 arc-second
	Slope		
	Aspect		
Land use /land cover	Proportion of water land (LU1)	Impact Observatory for Esri (public domain)	10 m
	Proportion of vegetation land (LU2)		
	Proportion of cultivated land (LU3)		
	Proportion of construction land (LU4)		
Building scale	Building height (BH)	Global Human Settlement Layer (GHSL), European Commission (EC), Joint Research Centre (JRC) (public domain)	100 m
	Building area (BA)	GHSL, EC, JRC (public domain)	100 m
Socioeconomic factors	Population count (PC)	GHSL, EC, JRC (public domain)	100 m
	Urbanisation degree (UD)	GHSL, EC, JRC (public domain)	1000 m

#### 2.2.2 Urban spatial morphology factor system

**Fig 2:**
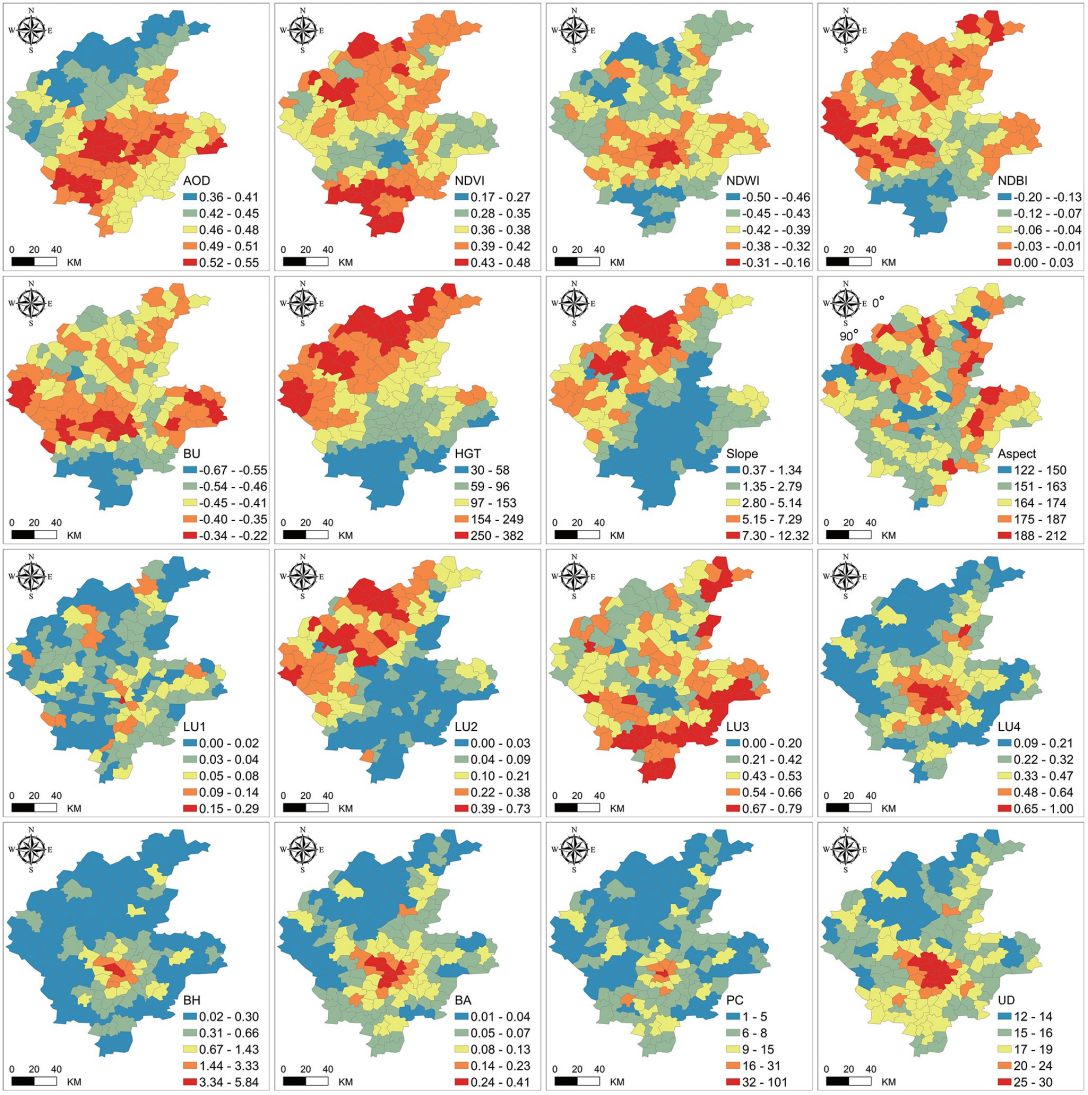
Spatial variation of explanatory variables related to LST. USGS EROS (Earth Resources Observatory and Science (EROS) Center) (public domain): http://eros.usgs.gov/; ESRI’s (public domain) (https://www.arcgis.com/home/item.html?id=cfcb7609de5f478eb7666240902d4d3d); European Commission’s Global Human Settlement Layer (public domain) (https://ghsl.jrc.ec.europa.eu/datasets.php).

Changes in industry, economy, culture, and public health affect the distribution of urban surface temperatures, leading to complex relationships between urban thermal environments and urban spatial characteristics [34]. This study collects six types of factors—Atmospheric Quality, Remote Sensing Indicators, Terrain, Land Use/Land Cover, Building Scale, and Socioeconomic Factors—comprising 16 variables (see Table 1) to construct an urban spatial morphology factor system [35–37]. Fig 2 illustrates the spatial distribution characteristics of the explanatory variables related to LST at the township scale.

Prior to using these explanatory variables, pre-detection processing is necessary to prevent significant multicollinearity issues among the variables. Table 2 lists the basic statistical information, including Min, Max, Mean, Skewness coefficient, Kurtosis coefficient, Pearson correlation coefficient, Global Moran’s Index, and Variance Inflation Factor (VIF) of the data for each variable. Results indicate that all variables have VIF values below 7.5, showing no significant multicollinearity.

**Table 2:** Statistical description of variables.

Variable	Min	Max	Mean	Skewness coefficient	Kurtosis coefficient	Pearson correlation coefficient	Global Moran’s I	VIF
AOD	0.27	0.61	0.46	-0.46	-0.27	0.65	0.75	2.54
NDVI	-0.30	0.77	0.38	-0.88	2.21	-0.28	1.11	7.02
NDWI	-0.72	0.54	-0.42	2.81	13.64	0.34	1.23	7.48
NDBI	-0.49	0.44	-0.05	-0.62	0.09	-0.26	0.78	2.82
BU	-1.20	0.30	-0.43	-0.29	-0.23	0.01	0.93	7.29
HGT	14.00	1134.00	155.32	1.66	4.00	-0.71	0.75	4.74
Slope	0.00	40.63	3.38	2.38	6.16	-0.62	0.69	5.12
Aspect	0.00	359.59	169.13	0.02	-1.10	-0.24	0.33	1.41
LU1	0.00	0.29	0.03	5.25	25.59	0.05	0.17	1.86
LU2	0.00	0.73	0.15	1.91	1.64	-0.59	0.57	6.82
LU3	0.00	0.79	0.52	-0.09	-1.99	0.11	0.95	6.06
LU4	0.09	1.00	0.29	0.93	-1.14	0.35	1.22	6.46
BH	0.00	31.68	0.35	5.00	38.59	0.30	1.45	6.84
BS	0.01	0.41	0.06	2.52	6.16	0.42	1.37	7.46
PC	1.06	100.58	5.73	6.80	101.99	0.19	0.91	3.09
UD	10.00	30.00	16.10	0.88	-0.40	0.45	1.31	3.75

### 2.3 Methods

**Fig 3:**
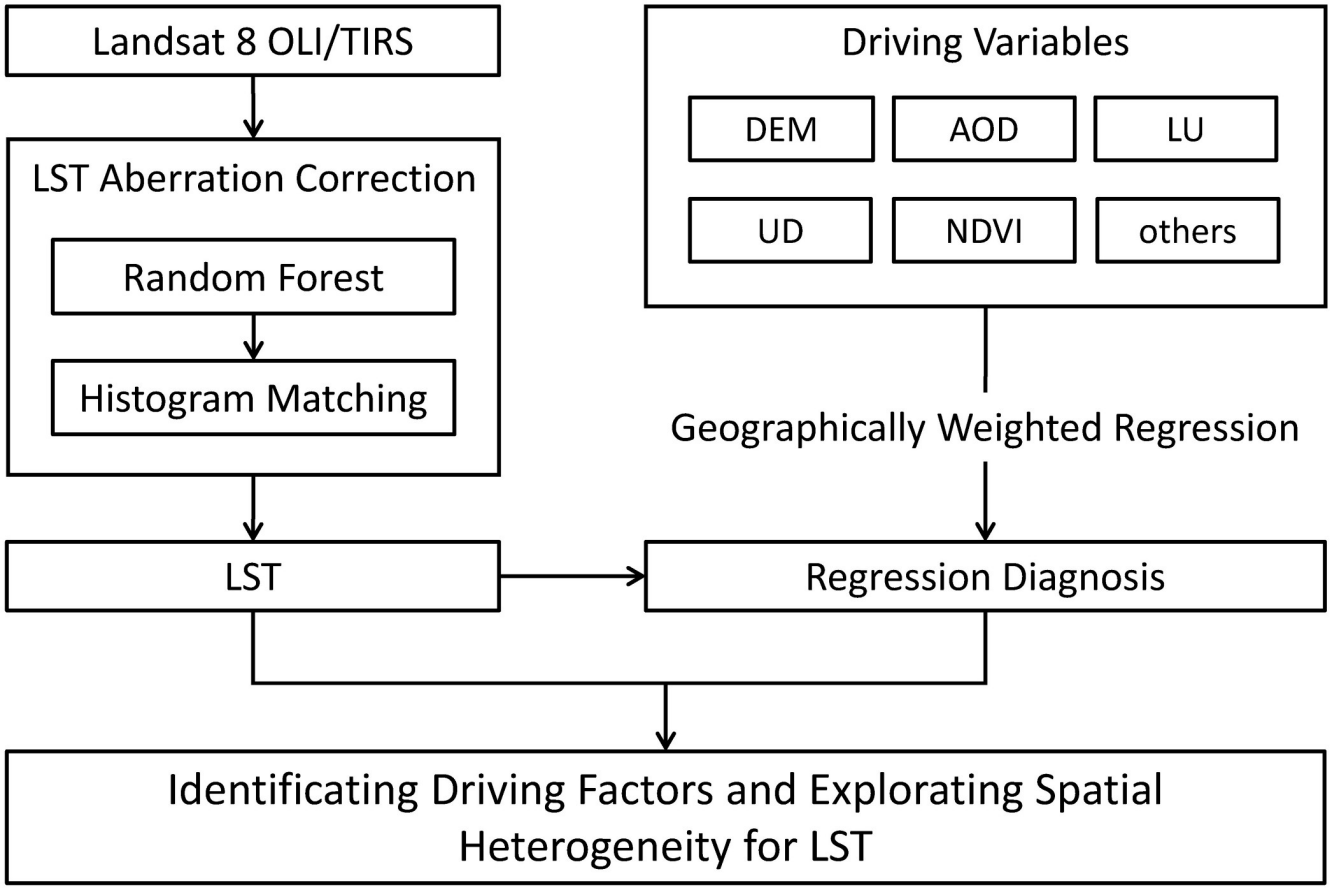
Flowchart of LST data processing and analysis of driving factors.

This study uses a township scale, based on Landsat remote sensing images to retrieve surface temperature, and explores the impact of urban spatial morphology on LST using spatial autocorrelation and spatial regression methods (Fig 3). First, a histogram equalization algorithm of random forests is used to calculate corrected surface temperature products from the radiance temperature and atmospheric parameters of the original satellite images. Then, variables related to surface temperature, such as aerosol optical depth (AOD), digital elevation model (DEM), land use (LU), urbanization degree (UD), and normalized difference vegetation index (NDVI), are selected as explanatory variables for spatial regression analysis. Finally, spatial regression methods (OLR, SEM, and GWR) are employed to establish spatial relationship models between surface temperature and explanatory variables, analyzing the spatiotemporal distribution characteristics and driving factors of surface temperature.

#### 2.3.1 LST data correction

Landsat surface temperature data cannot be used directly in scientific research and applications due to issues such as atmospheric correction, radiometric correction, surface feature correction, and striping problems [38]. These correction steps are necessary to eliminate atmospheric effects, ensure data accuracy and comparability, and fix striping issues in images, thereby improving data quality and reliability for scientific research and applications of surface temperature [39].

Random forests, an ensemble learning-based machine learning algorithm, can accomplish regression prediction tasks for Landsat surface temperature data [40]. First, raw Landsat surface temperature image data are preprocessed, extracting spectral and spatial features. The striping problem is set as a supervised learning target, and a model is established using the random forests algorithm. The model is trained with a training dataset to ensure it accurately identifies the target task features. The trained model is then used to predict new Landsat image data with striping issues, generating corrected image data.

In this paper, the measured daily LST data at a ground site (Daily data of meteorological station in Linyi City) is used to evaluate the LST inversion accuracy. Accuracy verification indicators include coefficient of determination (R^2^), root mean square error (RMSE).

#### 2.3.2 Spatial autocorrelation

In urban thermal environment studies, spatial autocorrelation describes spatial correlation in geographic data [38]. Moran’s I is a common spatial autocorrelation statistic, measuring the overall spatial autocorrelation degree of geographic spatial data [39]. It can be divided into global Moran’s I and local Moran’s I. The calculation formula is as follows:

$$Global\;Moran^{\prime }s\;I=\frac{n\;\sum_{i=1}^{n}\;\sum_{j\neq i}^{n}\;W_{ij}\left(x_{i}-\bar{x}\right)\left(x_{j}-\bar{x}\right)}{\sum_{i=1}^{n}\;\sum_{j\neq i}^{n}\;W_{ij}\;\sum_{i=1}^{n}{\left(x_{i}-\bar{x}\right)}^{2}}$$ (1)

$$Local\;Moran^{\prime }s\;I=\frac{n\left(x_{i}-\bar{x}\right)\sum_{j=i}^{n}\;W_{ij}\left(x_{j}-\bar{x}\right)}{\sum_{i=1}^{n}{\left(x_{i}-\bar{x}\right)}^{2}}$$ (2)

where n is the number of geographic units; x_i_ is the observed value of the ith geographic unit; $\bar{\text{x}}$ is the mean of all geographic units; W_ij_ is the spatial weight between geographic units i and j. Moran’s I ranges from -1 to 1, with positive values indicating positive correlation and negative values indicating negative correlation. Values close to 1 or -1 suggest significant clustering patterns.

Local Indicators of Spatial Association (LISA) aggregation analysis detects local spatial clustering patterns in geographic space. By calculating Moran’s I for each geographic unit and its neighbors, it identifies units with significant spatial clustering or dispersion. LISA aggregation analysis produces four quadrants: High-High (H-H), Low-Low (L-L), High-Low (H-L), and Low-High (L-H). H-H and L-L quadrants indicate clustering patterns, while H-L and L-H indicate dispersion patterns, helping to understand local clustering characteristics in spatial distribution.

#### 2.3.3 Spatial regression models

This study employs spatial regression analysis to investigate the impact of driving factors on LST spatial characteristics. Spatial regression models effectively address spatial dependence issues that linear regression analysis cannot handle. Common spatial regression models include the Spatial Error Model (SEM) and Geographically Weighted Regression (GWR).

The SEM utilizes spatial autoregression theory to reflect spatial dependence issues [40]. It measures the impact of spatial dependence in the disturbance error term on local observations. The SEM model formula is:

$$Y=X\beta +\lambda W_{\varepsilon }+\mu$$ (3)

where λ represents the spatial autoregressive coefficient of the error term; W_ε_ represents the spatial weight matrix; μ is the error term vector.

GWR is a regression analysis method for spatial data. Unlike traditional global regression models, GWR allows model parameters to vary spatially, better capturing heterogeneity and local associations in geographic spatial data [41]. The GWR model is expressed as:

$$Y_{i}=\beta_{i}^{0}+\sum_{k=1}^{P}\;\beta_{i}^{k}X_{i}^{k}+\varepsilon_{i}$$ (4)

where Y_i_ is the observed value of the dependent variable at location i; ${\text{X}}_{\text{i}}^{\text{k}}$ is the observed value of the kth independent variable at location i; $\beta_{\text{i}}^{0}$ and $\beta_{\text{i}}^{\text{k}}$ are the intercept and regression coefficients at location i; ε_i_ is the error term.

The key point of GWR is using different regression coefficients for each location i. The spatial weight matrix reflects the variation of spatial dependence within the study area, allowing GWR to detect spatial heterogeneity and improve regression fitting accuracy. GWR typically uses a specific distance decay function to determine the weight matrix elements, with spatial bandwidth controlling the decay rate. The closer the samples, the greater the weight, and the farther the samples, the smaller the weight.

To verify model accuracy, commonly used indicators like R^2^, RMSE, and Akaike Information Criterion (AIC) compare the results of the three global regression models. These indicators represent relative measures of statistical fit, where a better model has a higher R^2^ and lower AIC.

## 3. Results analysis

### 3.1 Temporal and spatial characteristics of LST

**Fig 4:**
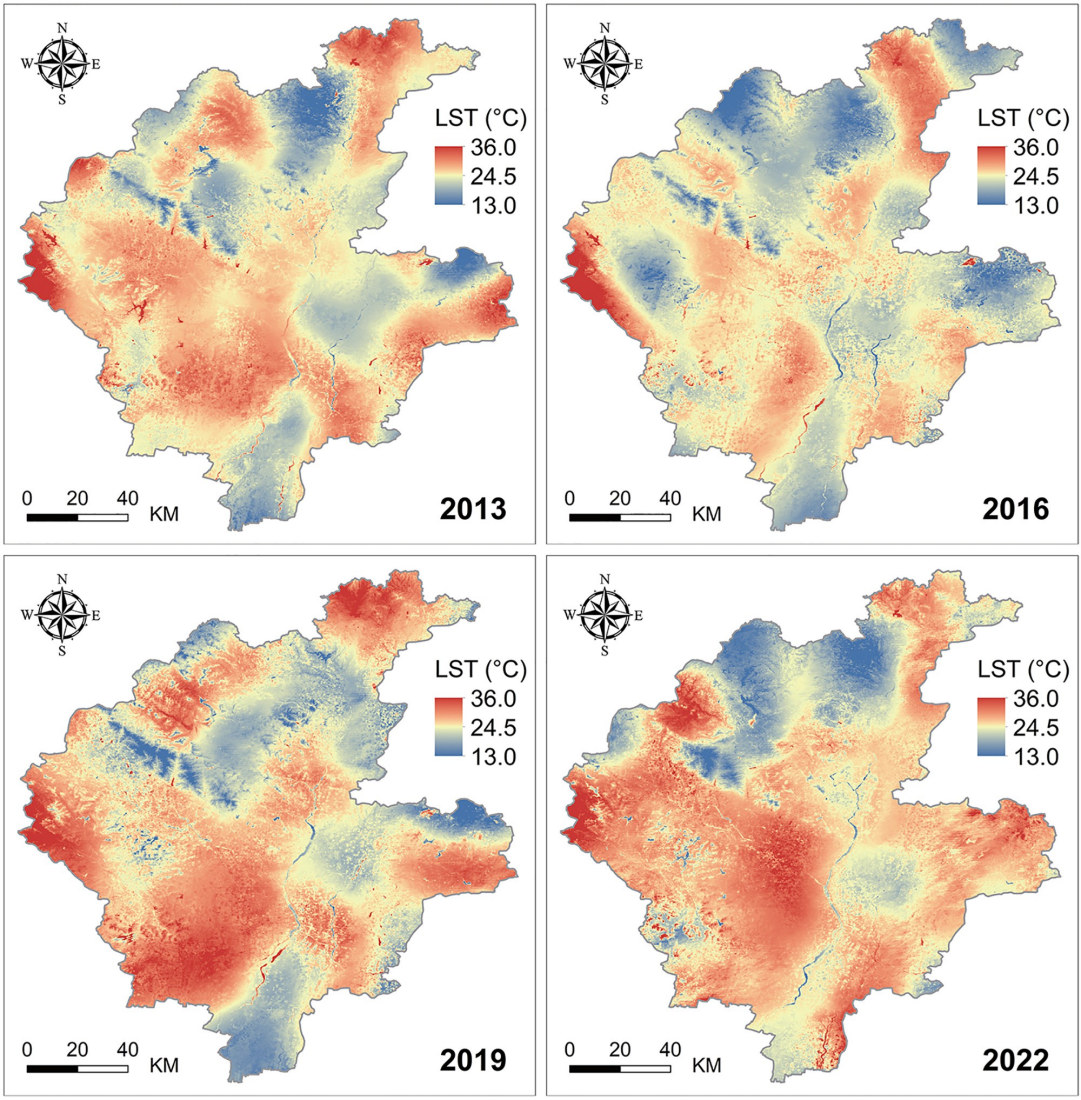
Temporal and spatial evolution patterns of LST in Linyi from 2013 to 2022. USGS EROS (Earth Resources Observatory and Science (EROS) Center) (public domain): http://eros.usgs.gov/.

Using the Random Forest Histogram Homogeneity Algorithm, we obtain a high accuracy LST result with an average R2 of 0.94 and an average RMSE of 3.31. We generated spatial distribution maps of LST for Linyi during four years from 2013 to 2022 (Fig 4). The annual average daytime LST in Linyi showed a rising trend from 2013 to 2019, followed by a slight decline. Geospatially, the surface temperature in southern towns was slightly higher than in the northern areas. The towering terrain of Mengshan at the border of Mengyin County and Pingyi County created a natural low-temperature zone, which was also observed in the western hilly areas of Yishui County. The Yi River, running through Linyi from north to south, served as a demarcation line for surface temperature, with the western side exhibiting significantly higher LST than the eastern side. Lanshan District, Luozhuang District, and Lanling County, characterized by flat terrain and dominated by urban land use, industrial development, and dense populations, generally had higher surface temperatures. In contrast, the northwestern mountainous areas, primarily covered by forests, had relatively lower surface temperatures.

**Fig 5:**
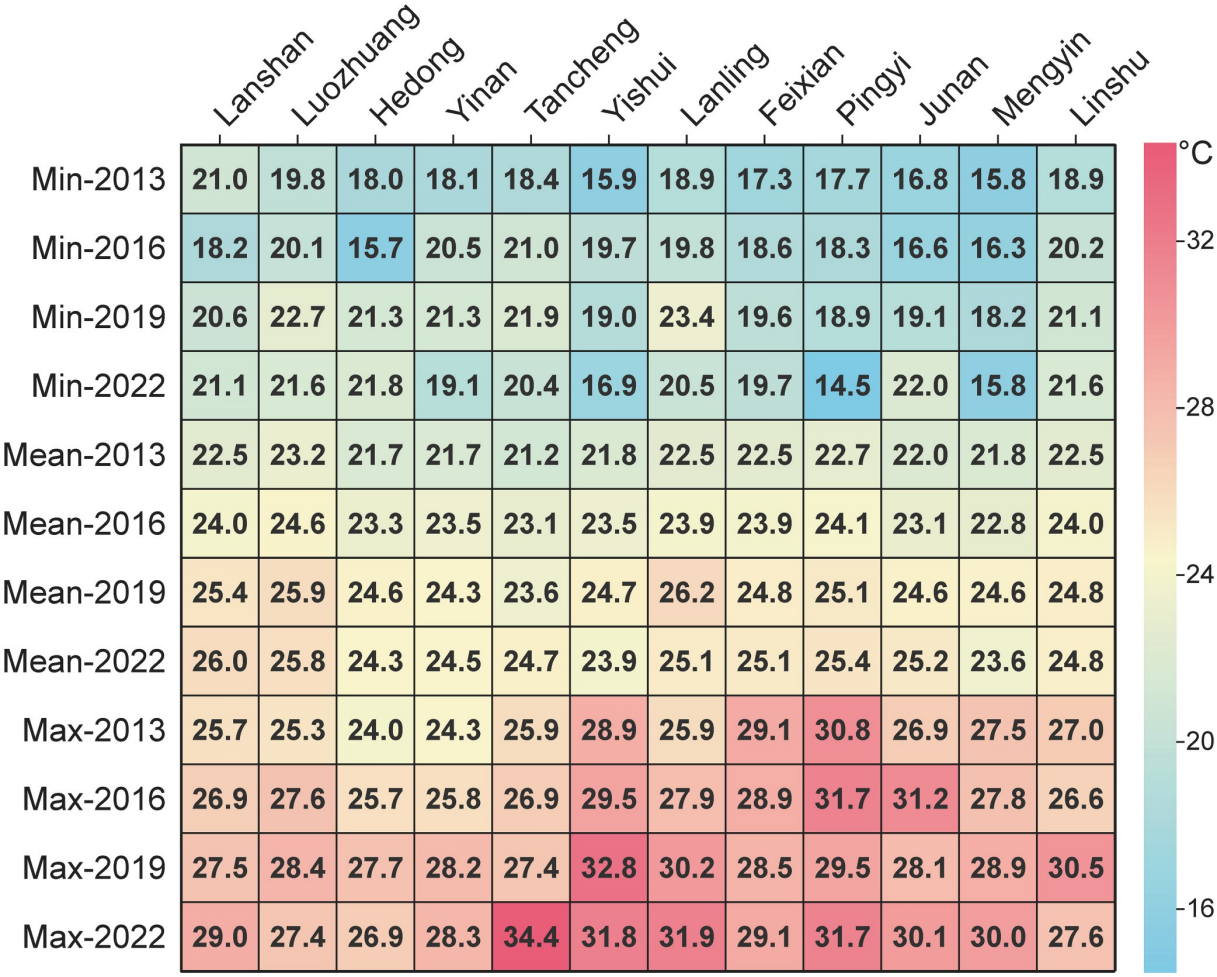
Statistical results of LST in various Counties and Districts of Linyi across four periods from 2013 to 2022.

A detailed spatial statistical analysis at the township scale was conducted for various counties and districts in Linyi, aiding in uncovering deeper spatiotemporal evolution patterns of the urban thermal environment. Fig 5 presents the minimum, average, and maximum surface temperatures for each county and district across four periods. The average temperatures in Luozhuang District, Lanshan District, and Lanling County remained consistently high, while those in Tancheng County, Yishui County, and Mengyin County were relatively low. Comparing the highest and lowest annual surface temperatures, Tancheng County in 2022 and Yishui County in 2019 recorded the highest temperatures at 34.4°C and 32.8°C, respectively; Pingyi County (2022) and Hedong District (2016) recorded the lowest temperatures at 14.5°C and 15.7°C, respectively. Overall, Lanling County, Lanshan District, and Tancheng County exhibited the most significant changes in average temperature, with maximum increases of 3.7°C, 3.5°C, and 3.5°C over nine years, respectively.

### 3.2 Spatial autocorrelation of LST

**Fig 6:**
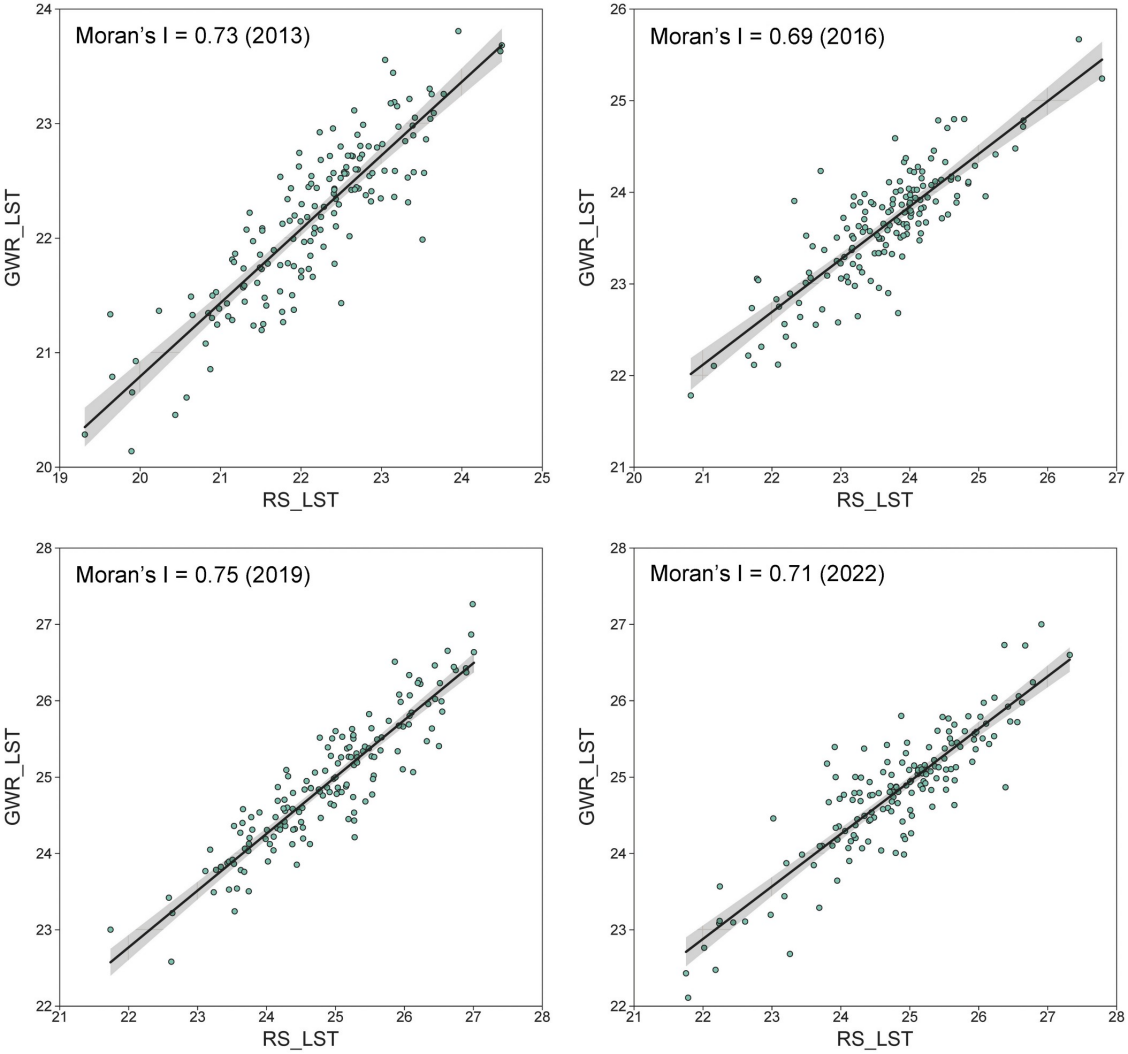
Moran scatter plots of LST in various Townships of Linyi from 2013 to 2022.

Global and local spatial autocorrelation analysis methods were employed to explore the spatial heterogeneity of surface temperature. Fig 6 displays the fitting relationships between the remotely sensed LST and GWR estimation results for the four periods, providing a foundation for quantifying the spatial variation of LST data. The results indicated that the global Moran’s I values for Linyi’s surface temperature from 2013 to 2022 were 0.73, 0.69, 0.75, and 0.71, respectively, demonstrating a significant positive spatial correlation.

**Fig 7:**
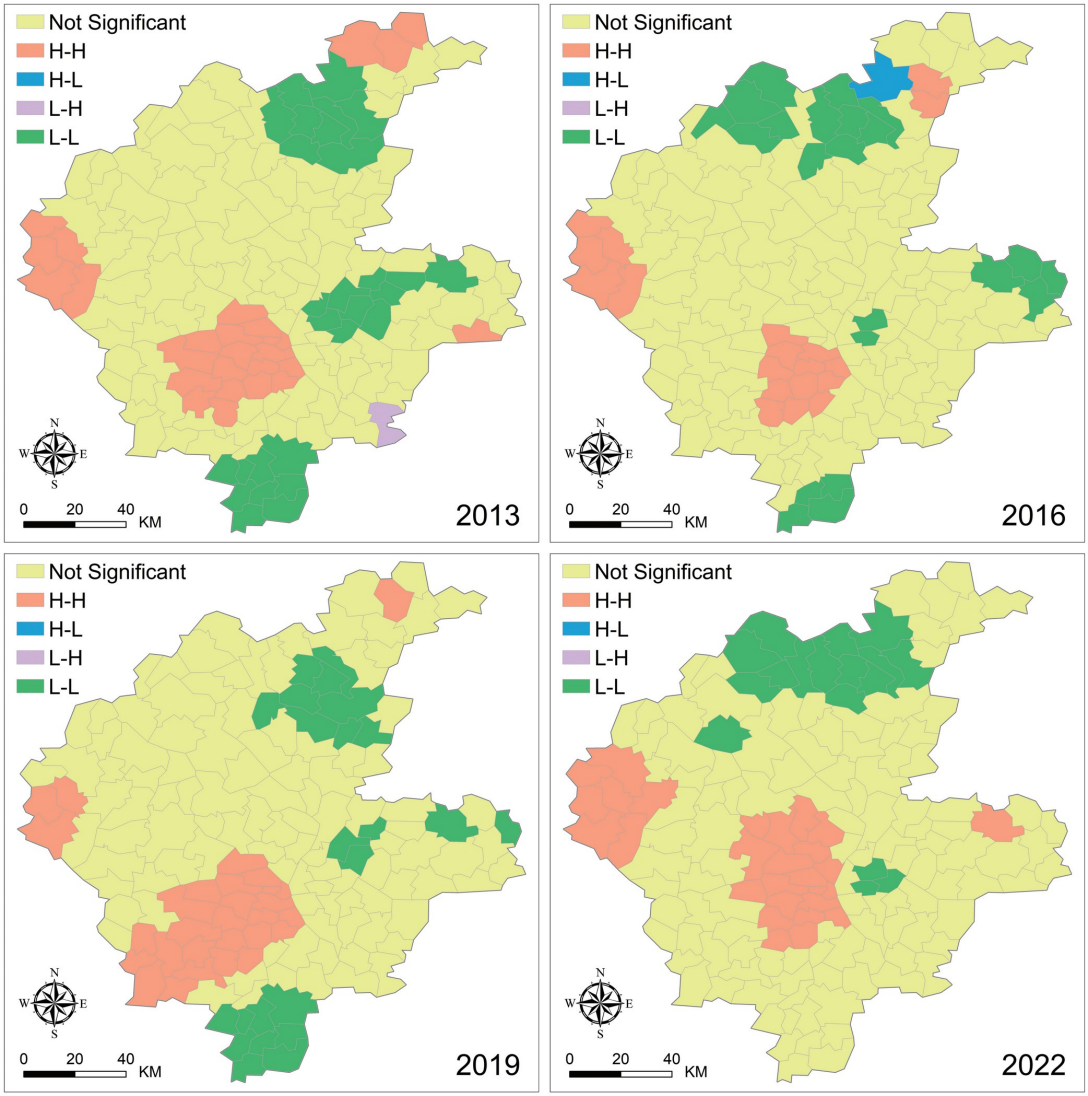
Spatiotemporal clustering patterns of surface temperature in various Townships of Linyi from 2013 to 2022. USGS EROS (Earth Resources Observatory and Science (EROS) Center) (public domain): http://eros.usgs.gov/.

The spatial clustering analysis produced LISA distribution maps for the four periods (Fig 7), effectively explaining the local spatial autocorrelation patterns of LST at the township scale. The spatial distribution patterns of surface temperature from 2013 to 2022 were generally similar. H-H clusters primarily occurred in Luozhuang District, Lanshan District, and Pingyi County, with occasional occurrences in Yishui County, Junan County, and Lanling County. L-L clusters were concentrated in Yishui County, with sporadic distributions in Tancheng County, Junan County, and Mengyin County. H-L and L-H clusters appeared in only a few years and a limited number of townships.

### 3.3 Regression results and influencing factors

To compare the regression performance of different models on surface temperature, MLR, SEM, and GWR models were used sequentially to fit the LST data. This study selected 16 explanatory variables for various townships in 2022 as independent factors, with the corrected surface temperature as the dependent variable. Before conducting the regression experiments, all variables were cleaned to correct their distribution and eliminate outliers, enhancing the explanatory power of the regression models.

Table 3 presents the regression analysis results of MLR, SEM, and GWR models, with evaluation metrics including R^2^, adjusted R^2^, RMSE, and AIC. Compared to MLR and SEM, the GWR model achieved higher R^2^ and adjusted R^2^, while RMSE and AIC were lower. The values for these four metrics were 0.681, 0.645, 0.595, and 334.368, respectively. Evidently, the GWR model outperformed MLR and SEM in fitting accuracy, thus it was chosen to explain the regression relationships among variables in this study.

**Table 3:** Comparative results of regression analysis using MLR, SEM, and GWR models.

Type	Variable	MLR	SEM	GWR
	Constant	19.813	19.733	16.313
Atmospheric quality	AOD	6.101	6.101	6.782
Remote sensing indicators	NDVI	-16.312	-16.312	-10.992
	NDWI	-9.916	-9.916	-5.093
	NDBI	12.461	12.461	16.058
	BU	-7.499	-7.499	-8.839
Terrain	HGT	-0.120	-0.131	-0.172
	Slope	0.293	0.303	0.544
	Aspect	0.846	0.853	0.601
Land use /land cover	LU1	-0.081	-0.070	-0.042
	LU2	-0.113	-0.125	-0.196
	LU3	-0.043	-0.043	0.049
	LU4	-1.792	-1.782	-1.157
Building scale	BH	-1.286	-1.286	-0.613
	BA	3.263	3.263	1.913
Socioeconomic factors	PC	0.279	0.279	0.159
	UD	0.740	0.740	1.470
	R^2^	0.401	0.413	0.681
	Adjusted R^2^	0.333	0.340	0.645
	RMSE	0.816	0.806	0.595
	AIC	422.273	383.963	334.368

**Fig 8:**
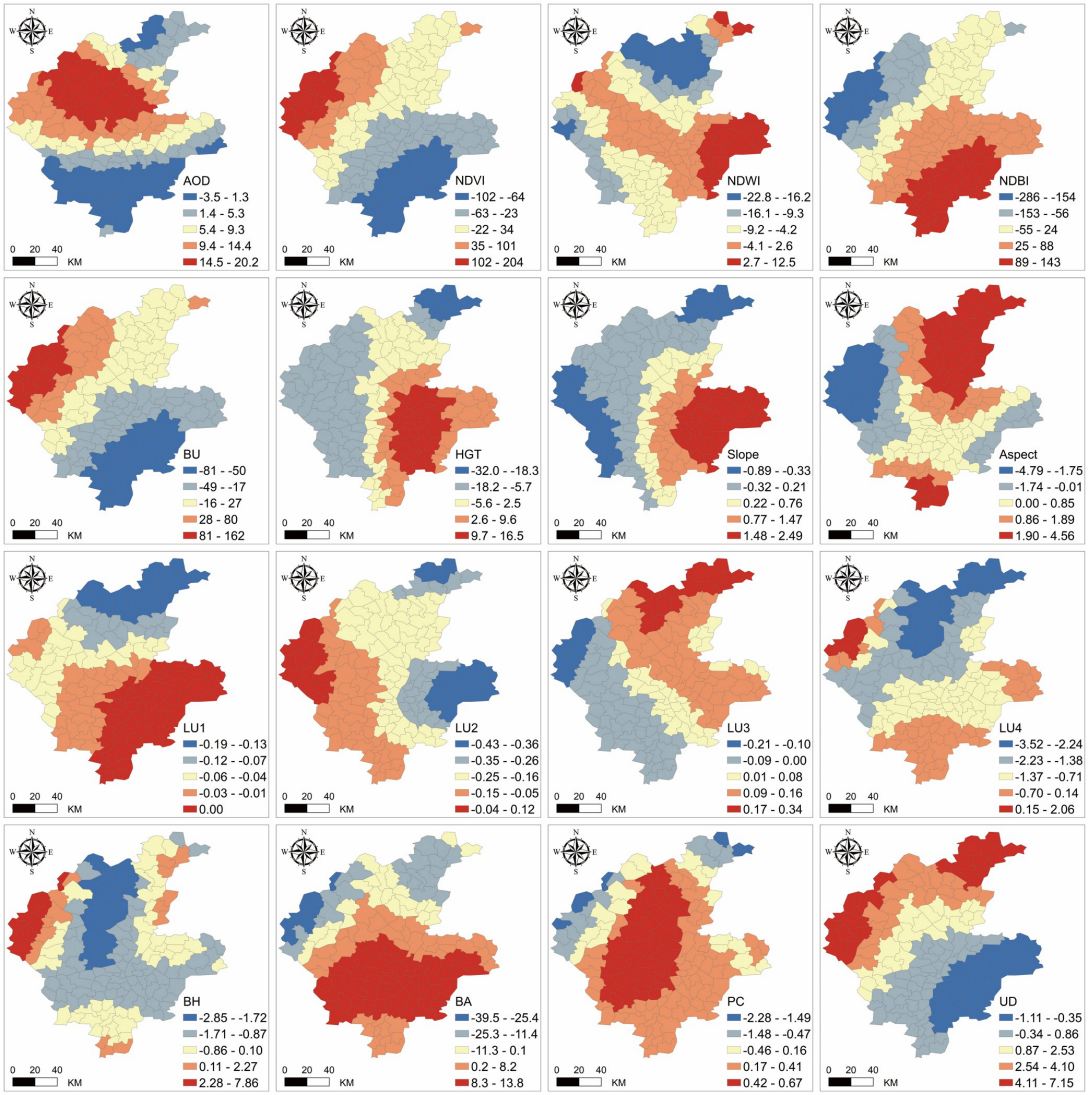
Spatial heterogeneity characteristics of 16 influencing factors over Linyi in 2022.

Fig 8 illustrates the spatial variability of regression coefficients for 16 driving factors in Linyi in 2022. All variables exhibited significant spatial heterogeneity, presenting a stratified state spatially. The positive influence of AOD on LST was mainly concentrated in the hilly areas north of the Mengshan mountain range, at the intersection of Mengyin County, Yinan County, and Yishui County. The spatial distribution of the BU regression coefficient in remote sensing indices was contrary to NDBI but very similar to NDVI. HGT and Slope in topographic features were similar, with some differences in the eastern part of Junan County. The spatial performance of the regression coefficients for the four land use types was normal, showing higher positive effects in areas with dominant land use types and more pronounced negative effects in regions with less land use type distribution. The high values of BH regression coefficients in building scale generally appeared on the outskirts of Linyi, particularly to the west of Pingyi County; the BA regression coefficients significantly influenced most areas of Lanling County, Fei County, Lanshan District, Luozhuang District, Hedong District, Linshu County, and Junan County. Among socioeconomic factors, the positive effect of PC was mainly in central Linyi, while UD showed a trend of gradually changing from positive to negative from northwest to southeast.

## 4. Discussion

Previous studies have primarily focused on indicators related to landscape patterns, building forms, and surface parameters to comprehensively analyze how urban form influences UHI from both 2D and 3D perspectives [41]. However, the impact of the urban heat island effect can extend across the entire urban area, including remote rural regions and even adjacent cities, often overlooking the systematic spatial morphology of cities [42]. We emphasize regional complexity from a geographical perspective, and constructs an urban spatial morphology factor system. Changes in air quality, topography, land use, remote sensing indices, and built-up area characteristics within a city all feedback into urban surface temperatures [43]. In addition, temporal resilience focuses on the dynamics, adaptability, and recovery capacity of cities in long-term evolution, emphasizing the sustained and progressive development of urban sustainability. The temporal resilience of urban sustainability requires an organic integration of urban planning, social, economic, technological, and ecological systems. Based on the above ideas, we preliminarily determined that topography, air quality, land use types, socioeconomic conditions, and building scale are more significant than remote sensing indices. Spatial autocorrelation and spatial regression methods were used to investigate the impact of urban spatial morphology on LST at the township scale.

**Fig 9:**
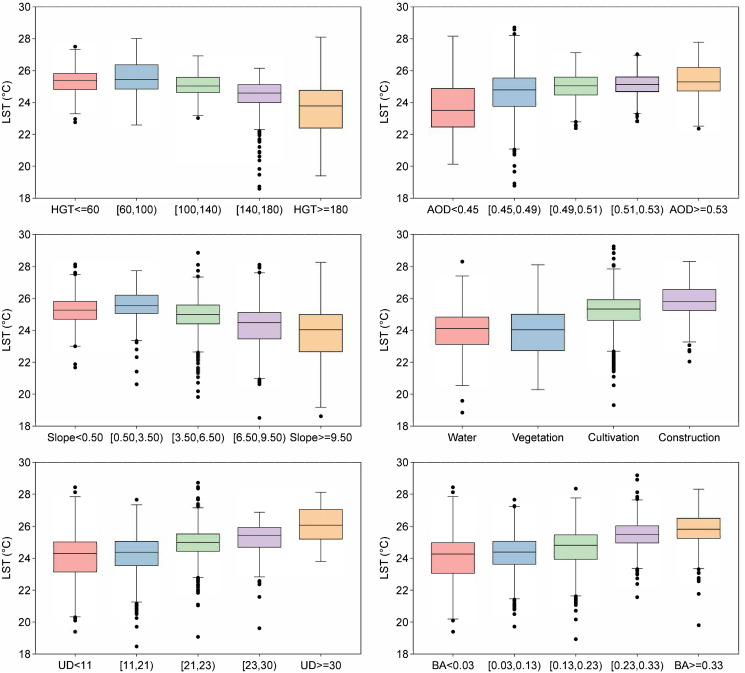
Boxplot of the impact of the main drivers on the LST in 2022.

The Boxplot of the impact of the main drivers on the LST is shown in Fig 9. Land use types, including water bodies, vegetation, cropland, and built-up land, were integrated into a single indicator with four subcategories, and their surface temperatures were analyzed collectively. Above 60 meters, higher HGT corresponded to lower LST; AOD showed a positive correlation with LST; above 0.5, steeper slopes corresponded to lower LST; water bodies and vegetation had the lowest LST, followed by cropland, with built-up land having the highest LST; higher UD corresponded to higher LST; BA showed a positive correlation with LST. These six factors drive LST changes in different ways, thereby affecting the urban heat island effect.

This study used remote sensing data and other geographical data, employing spatial statistical methods and spatial autocorrelation analysis to explore the spatiotemporal differentiation characteristics and driving factors of surface temperature at the township scale. However, several limitations and deficiencies remain in the study content and methods. (a) Due to the limitations of data acquisition frequency, it was impossible to obtain explanatory variables for all periods, making the use of single-period variables insufficient for real-time explanations of surface temperature. (b) The study only discussed the spatial scale at the township level, neglecting the errors in spatial heterogeneity expression of driving factors at different spatial scales. (c) The obtained surface temperature data were annual daytime data, without considering the interference of differences at different temporal scales (day and night, monthly, seasonal, etc.) on the spatiotemporal characteristics of surface temperature [44]. Future research should focus on multi-level temporal scales, multi-level spatial scales, and higher real-time multi-source data, jointly using global statistical methods, local statistical methods, and spatiotemporal statistical methods to comprehensively explore the spatiotemporal characteristics and driving factors of surface temperature.

## 5. Conclusion

This study described urban spatial morphology from six dimensions: spatial morphology, land use, landscape scale, socioeconomic conditions, topography, and remote sensing indices. It then used spatial autocorrelation and spatial regression models to study the spatiotemporal patterns of LST and its relationship with urban spatial morphology. From 2013 to 2022, the LST of all towns in Linyi showed a gradual increasing trend. Areas with high LST values were concentrated in the eastern and southern parts of the study area, characterized by flat terrain and primarily used for urban construction. Low LST values were concentrated in the northern and western areas, mainly influenced by mountainous topography and primarily covered by forests. From the perspective of global spatial autocorrelation, Moran’s I values for LST in the four periods were relatively high, indicating significant positive spatial correlation. In terms of local spatial autocorrelation, high-high LISA values were distributed in the central and western areas, while low-low LISA values were scattered in the northern parts and some isolated counties. The spatial characteristics of LST are significantly influenced by urban spatial morphology, with the strongest correlations found in the factors of land use types, landscape metrics, and remote sensing indices, followed by socioeconomic characteristics. Compared to MLR and SEM methods, the GWR model achieved the best results in analyzing the influence of driving factors on LST, due to its consideration of spatial non-stationarity, yielding the highest fit. The findings aim to provide valuable references for town planning, resource allocation, and sustainable development.

## Supporting information

S1 File Supplementary data to this article can be found in supplementary material. (ZIP)
